# Light-PTNet: A lightweight parallel temporal network for smartphone-based human motion classification

**DOI:** 10.1371/journal.pone.0331135

**Published:** 2025-09-23

**Authors:** Sarmela Raja Sekaran, Pang Ying Han, Ooi Shih Yin, Lillian Yee Kiaw Wang, Lim Zheng You

**Affiliations:** 1 Faculty of Information Science and Technology, Multimedia University, Malacca, Malaysia; 2 Centre for Advanced Analytics, CoE for Artificial Intelligence, Multimedia University, Malacca, Malaysia; 3 School of Information Technology, Monash University, Selangor, Malaysia; Sunway University, MALAYSIA

## Abstract

The increased popularity of smartphone-based human activity recognition (HAR) in recent decades has been driven by its low computational requirements and user privacy protection. Yet, developing a reliable smartphone-based HAR still presents several challenges. For example, handcrafted feature-based approaches highly depend on laborious feature engineering/selection techniques that require human intervention. Implementing conventional Convolutional Neural Networks may result in unsatisfactory performance in time series classification as they cannot effectively extract time-dependent features. Although recurrent models excel at extracting temporal information, they require extensive computational resources to attain high performance, limiting their practicality for real-time applications. Thus, we propose a lightweight smartphone-based HAR architecture called Lightweight Parallel Temporal Network (Light-PTNet) for reliable classification. Light-PTNet comprises parallelly organised Light Spatial-Temporal Heads (LSTC Heads) that capture underlying patterns at various scales of the inertial signals. These heads utilise dilations and residual connections to preserve longer-term dependencies without increasing the model parameters. This work assesses the proposed Light-PTNet’s performance on open-access HAR datasets: UCI HAR, WISDM V1, and UniMiB SHAR, following a user-independent protocol. The results reveal that our proposed Light-PTNet achieves 98.03% accuracy on UCI HAR, 81.58% on UniMiB SHAR and 97.02% on WISDM V1 with fewer model parameters (lower than 0.1 million parameters).

## 1. Introduction

Analysing and identifying human motions and behaviours is a prevalent task in today’s world, as this task has led to numerous advancements in various domains such as activity monitoring, health care, assisted ambient living, interactive games, etc. [1–4]. Generally, human activity recognition (HAR) systems are classified depending on the input data type and corresponding sensors used: visual-based and sensor-based HAR. Cameras are utilised for data collection in visual-based HAR, whereas sensor-based HAR utilises motion sensors, namely accelerometers, magnetometers and gyroscopes. Sensor-based HAR has a considerable advantage over visual-based HAR, as its performance is unaffected by environmental factors such as background noises (i.e., non-human motion), varying lighting conditions and occlusion. Moreover, sensor-based HAR systems uphold the user’s privacy as the user identification information, including images/videos of the user performing the activities, is not included in the collected inertial signals.

In recent years, due to rapid technological advancements, smartphones and smartwatches have been integrated with various motion sensors (e.g., accelerometers, gyroscopes, and magnetometers), which can capture data about people’s movement patterns. Hence, these smart devices have become favourable data-collecting tools in the HAR domain due to their lower cost, smaller size, flexible deployment, and user privacy features. The data collection process in smartphone-based HAR is unobtrusive and more convenient for users because they do not need to wear multiple sensors around their bodies while performing activities.

Researchers have devised various pattern analysis techniques for effective HAR. In the past, numerous HAR models were built based on handcrafted feature-based approaches due to the shortage of computational capacity. Handcrafted feature-based approaches require manual feature engineering to extract information from the signal input and classify those features using classical machine learning classifiers, namely AdaBoost [5], support vector machine (SVM) [6], Random Forest (RF) [7], Hidden Markov Model [8], *K*-Nearest Neighbour (KNN) [9], etc. However, nowadays, more deep learning approaches are extensively explored and developed to build HAR systems due to their exceptional performance. Further, the proliferating growth of computing power is also one of the factors of the numerous innovations in deep learning. The widely implemented deep learning models in the HAR domain include but are not limited to Convolutional Neural Networks (CNN) [10,11], Long Short-Term Memory Networks (LSTM) [12,13], Temporal Convolutional Networks (TCN) [14,15], etc.

### 1.1. Related work

In the past, handcrafted feature-based techniques dominated the HAR domain due to the scarcity of computational resources. Anguita et al. published a self-collected smartphone-based HAR dataset (i.e., UCI HAR) and performed statistical feature analysis on them to identify salient features [16]. The computed 562 features are further processed by the SVM classifier for data analysis and classification. In the work by Kolosnjaji et al., inertial input signals are transformed using the Fast Fourier Transform (FFT) before passing them into RF for classification [7]. Besides that, Kee et al. also proposed a handcrafted feature-based HAR solution by applying multiple feature selection techniques that determine the optimal features [17]. Correlation-based feature selection subset evaluator, correlation-based attribute evaluator, and information gain attribute evaluator are the chosen techniques to identify the relevant features before applying the RF classifier. In addition, Continuous Hidden Markov Model (CHMM) is proposed to recognise human activities by Ronao et al. [18]. There are two parts to CHMM: the first part of CHMM classifies the input samples as either static or dynamic activities, and the second part performs course classification where the static sample will be classified into sitting, standing or laying, and the dynamic sample will be classified into jogging, walking or climbing stairs. A promising classification performance is reported. Nonetheless, the computed features overly rely on domain knowledge in these methods, leading to low model generalising abilities.

Over the years, deep learning models have started gaining substantial attention due to their exceptional recognition performance. Ignatov et al. developed a simple multi-layered CNN network and incorporated manually extracted statistical features as additional information [10]. In addition, the authors applied the data centering technique on the input signals to boost the classification model accuracy by ~1.5%. Besides, Peppas et al. also devised a similar architecture, a CNN-based model [19]. The difference between these two models is the parameter configurations, where Peppas et al. employed many large convolutional kernels and hidden layer’s neurons in their architecture [19]. Huang et al. designed a deep learning network with three convolutional layers and employed a cross-channel communication block to maintain the data flow throughout the network [20]. This block comprises an encoder to capture all channel feature responses, a message-passing module to ensure the interaction between all channels, and a decoder to gather information from the corrected channels and transform the output into the desired dimension. This CNN model achieves relatively high classification accuracy across several databases, including UCI HAR, UniMiB SHAR, PAMAP2 and WISDM V1, with considerably low model parameters. In another work, Huang et al. replaced standard convolutional operations with channel-selective convolutional operations to develop a lightweight HAR model [21]. The channel-selective convolutional operation replaces useless or redundant channels with significant channels and counteracts the loss of diversity in the network due to numerous similar channels through channel deallocation, reallocation, and spatial shift. With only 0.33 million learnable parameters, the baseline model with channel-selective convolutional is able to achieve an accuracy of 96.77%. Although CNN architectures are adopted in modelling a HAR system, they are considered subpar in time series analysis as they do not capture temporal information effectively.

On the other hand, recurrent models are good at capturing temporal features and perform well in time series classification problems, especially motion analysis. Several researchers employ recurrent networks, such as LSTM, in the smartphone-based HAR domain to analyse temporal features and identify human activities. Mutegeki et al. integrated a CNN network with an LSTM to enable spatial and temporal feature extraction from the input [13]. The authors claimed that the amalgamation of these networks improved the classification performance with at least 1% accuracy. Mekruksavanich et al. adopted LSTM network to capture underlying patterns from input signals, and employed the XGB classifier to classify the extracted data [22]. Besides, Yu et al. presented a bidirectional LSTM model to classify input samples into respective activity classes [23]. Unlike the conventional LSTM where the network only predicts based on past information, this model could make predictions based on the input signals’ past and future information. Hence, richer information is obtained in this enhanced LSTM model. Zhao et al. presented a recurrent network called Deep Residual Bidir-LSTM and enhanced its performance by incorporating residual connections [12]. This incorporation reduces the effects of gradient vanishing and exploding issues. This network outperforms the ordinary bidirectional LSTM by ~2% accuracy score. Though conventional recurrent architectures improve classification accuracy, the high computational complexity and resource requirements remain major concerns.

Unlike recurrent architectures, TCN models are designed to perform data analysis and classification without demanding extensive computational resources. Additionally, this architecture facilitates the extraction of longer time-dependent features, which are crucial for analysing human actions and motions. Lea et al. developed TCN architectures to solve action segmentation problems [24]. The authors presented two variants of TCNs: one with an encoder and decoder module (ED-TCN) and another with convolutional blocks with dilations (Dilated TCN). Nair et al. utilised these models in the human activity recognition domain [14]. Empirical results demonstrate that these TCN-based architectures can attain higher accuracies with relatively lower computations. The authors observed that ED-TCN performed better than Dilated TCN, with a slight increase in accuracy by 1%. Garcia et al. further enhanced the TCN by proposing two TCN variants: one with an additional fully connected network (TCN-FCN) and another with an additional deep convolutional block (deepConvTCN) [25]. Empirical results show that these TCN-variant models could achieve higher accuracy than the ordinary TCN models. Nevertheless, to further boost performance, TCN models need to be deeper and denser. Garcia et al. and Nair et al. addressed this by implementing multiple stacks of convolutional layers with many large filters to build a denser model for the HAR task [14,25]. This design inadvertently raises the total learnable parameters, escalating the complexity of the computations and making the model more susceptible to overfitting problems.

### 1.2. Motivations and contributions

Despite the development of numerous HAR approaches throughout the years, there is still room for improvement in motion signal analysis due to the limitations of the existing methods. The limitations are as follows:

1. In handcrafted feature-based approaches, domain experts are expected to devise laborious and tedious feature engineering techniques, and the quality of feature engineering and selection techniques determines the success of these approaches. Since feature engineering is based on prior knowledge, the model may be susceptible to biases, leading to a poor generalising model.
2. CNN architectures are subpar in identifying temporal patterns.
3. Recurrent Neural Networks (RNNs) are vulnerable to vanishing and exploding gradient problems, where the network is unable to retain longer-term dependency. Although LSTM solves gradient issues, they are computationally expensive [26].
4. Despite having relatively low computations compared to recurrent models, improving TCNs’ performance necessitates the model to be denser and more complex [14,25].

**Fig 1:**
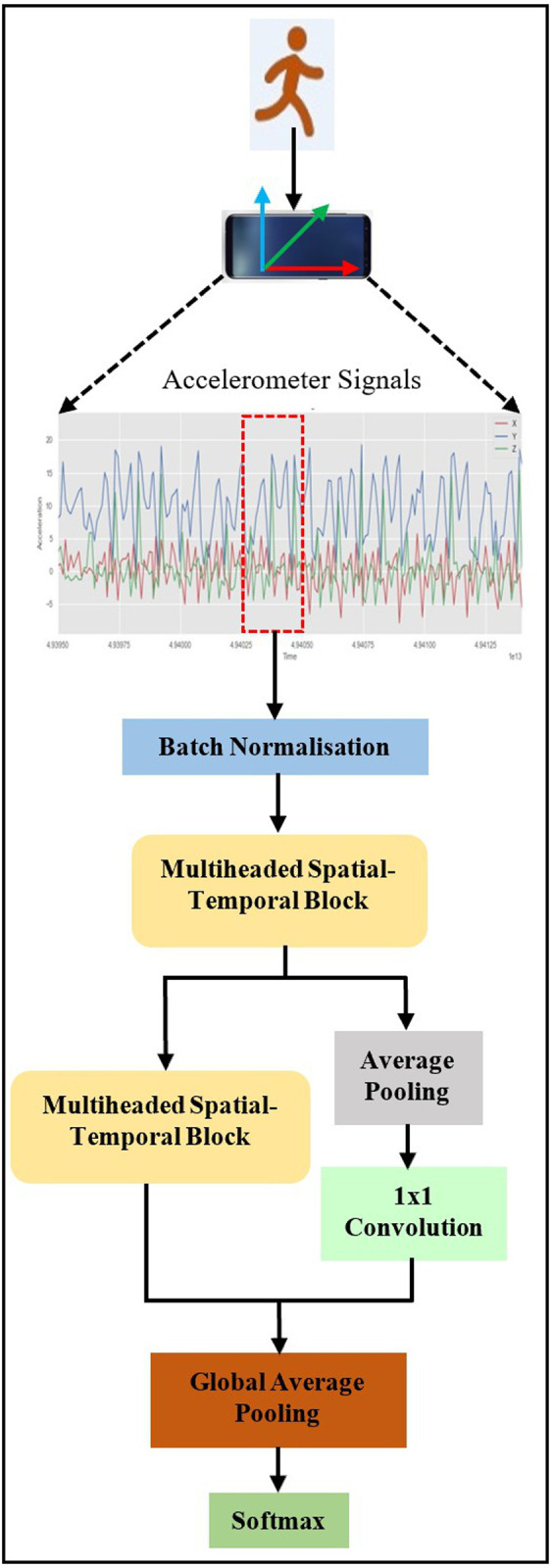
Overall proposed architecture.

In this paper, a lightweight TCN-based model, known as Light-PTNet, is proposed. Fig 1 illustrates the framework of the proposed Light-PTNet. Light-PTNet comprises core components, including Multiheaded Spatial-Temporal Blocks (MST Block) to perform multiscale feature extraction, average pooling to lower trainable parameters and computational complexity, Global Average Pooling (GAP) layer to minimise model overfitting and lastly, an output dense layer with softmax activation for classification. These collective components contribute to the efficiency and effectiveness of the models in feature extraction, hyperparameter minimisation and classification accuracy.

This research’s contributions are summarised as follows:

1. Light-PTNet leverages dilations and residual connections architectures to capture more extended deep spatial-temporal features with its exponentially extendable receptive field without suffering from vanishing or exploding gradient problems, particularly when training on long input inertial sequences. Furthermore, the proposed model can model longer-term dependencies by preserving a more extended effective history of the input motion signals.
2. Light-PTNet achieves multiscale spatial-temporal feature extraction by engaging three Light Spatial-Temporal Convolutional Heads (LSTC Heads) organized in parallel inside MST Blocks. Each head adopts different convolutional kernel sizes. Concatenating the extracted deep hierarchical features provides richer information for classification.
3. Light-PTNet implements one-dimensional separable convolutions instead of standard convolutional operations in the MST Blocks to ensure the proposed architecture is lightweight in computation. These convolutions drastically lower the trainable model parameters, reducing the computational complexity and cost.
4. The feasibility of the proposed Light-PTNet is verified using publicly accessible HAR databases (i.e., UCI HAR, WISDM VI, and UniMiB SHAR). The performance assessment is conducted under a user-independent procedure to evaluate the model’s generalisability across diverse user profiles.

This paper comprises five sections, and the following is the organisation of this paper:

- Section 1 provides the background study of the HAR domain, existing HAR approaches, challenges in developing HAR models and our work’s contributions.
- Section 2 describes the proposed Light-PTNet designed for the HAR task. The components and the functionality of the proposed architecture are explained in detail.
- Section 3 presents experimental setups, such as the evaluation dataset descriptions and model configurations implemented throughout this work.
- Section 4 discusses the proposed Light-PTNet’s architectural analysis, performance and computational complexity comparison of Light-PTNet and existing HAR models.
- Section 5 summarises this work’s contributions.

## 2. Proposed method

Light-PTNet’ core architecture is inspired by Dilated TCN since this model is specifically designed for time series classification tasks, while sensor-based motion data is represented as a time series. Hence, it is pertinent to analyse human activity inertial data. The benefits of adopting Dilated TCN architecture are as follows:

i TCNs accept input sequences with variable lengths and can generate outputs with the same length as inputs.
ii Implementing the same filters in each layer enables parallel executions of convolutional operations, ensuring TCNs can parallelise better [26].
iii TCNs have the ability to extend kernels’ field of view without raising the trainable parameters due to dilations [27].
iv TCNs retain a longer-term dependency by incorporating dilated convolutions and residual connections.
v Furthermore, residual connections allow these models to have stable gradients, greatly reducing the effects of vanishing and exploding gradients [28].
vi Lastly, the memory requirement is relatively low while training a TCN, as this model does not perform gate operations nor store partial results throughout the network.

**Fig 2:**
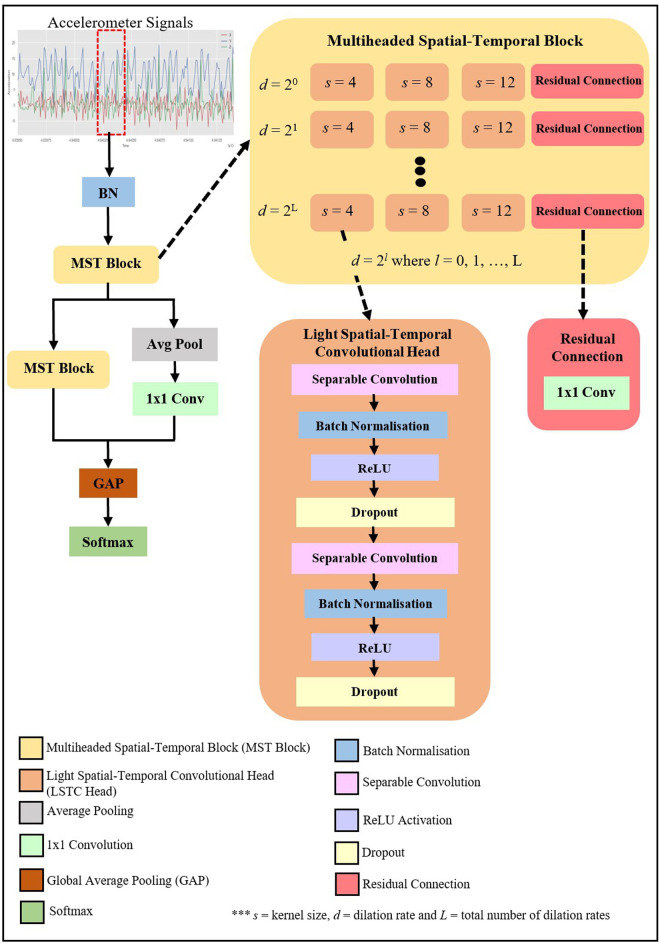
The detailed architecture of Light-PTNet.

Fig 2 illustrates the architecture of the proposed Light-PTNet. The input inertial signals are segmented and forwarded to the batch normalisation (BN) layer. Then, the normalised input is fed into MST Blocks. MST Block has multiple LSTC Heads with different dilation rates and kernel sizes for multiscale feature extraction. Each Separable Convolutional Head comprises two sets of one-dimensional (1D) separable dilated convolution, batch normalisation, ReLU activation and dropout. After the first MST Block analysis, the output will be passed to the subsequent MST Blocks as well as an average pooling (Avg Pool) layer, followed by a one-by-one (1 × 1) convolutional layer. The outputs of the second MST Block and the 1 × 1 convolutional layer are concatenated before passing them to the GAP and softmax layer. The following subsections further explain the proposed Light-PTNet’s implementation details.

### 2.1. Multiscale feature extraction

The core constituent in the proposed model is the MST Blocks. Essentially, MST Blocks allow multiscale feature extraction. MST Block’s architecture is illustrated in Fig 2 (dark yellow box). The MST Blocks have multiple LSTC Heads (orange box) with different dilation rates and kernel sizes. This design facilitates multiscale feature extraction. In each dilation level with d rate, there are three LSTC Heads and one residual connection. Each LSTC Head comprises two sets of separable dilated convolution, batch normalisation, ReLU activation, and dropout regularisation, as shown in Fig 2 (orange box). Different convolutional kernel sizes, i.e., 4, 8, and 12, are adopted in the LSTC Heads. Specifically, the proposed Light-PTNet simultaneously implement convolution operations with different-length kernels in each LSTC Head at each dilation level to perform multiscale feature analysis. In other words, this multiscale feature extraction process enables the model to extract features at diverse scales, precisely identifying the underlying patterns of the dynamic inertial signals. In contrast, every dilation level in a conventional TCN has only one fixed-sized receptive field. Thus, there is no multiscale feature extraction at each level.

In LSTC Head, the generated feature maps from each separable dilated convolution will go through batch normalisation, ReLU activation and dropout regularisation. Batch normalisation minimises the model’s internal covariate shift (i.e., the distribution of every subsequent layer’s input constantly changing in line with the earlier layers’ parameters). As the model goes deeper, the effects of internal covariate shift increase; thus, this can trigger a slower model convergence during training. Hence, batch normalisation reduces the effect of this phenomenon by introducing two new learnable parameters that determine the optimal values for scaling and shifting each normalisation’s distribution curve. The proposed model applies a dropout layer after every activation function to prevent the model from overfitting. Before passing the extracted feature maps to the subsequent layers, element-wise addition concatenates LSTC Heads’s outputs with the residual connection’s output.

**Fig 3:**
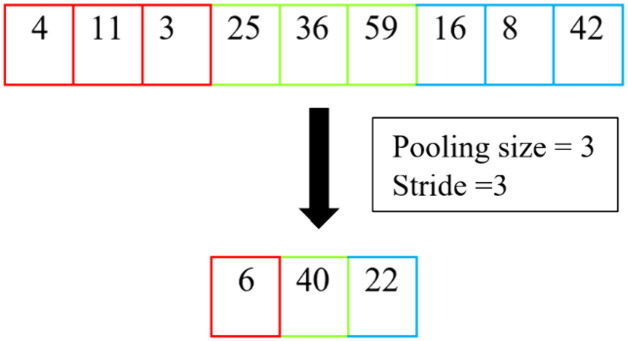
Average pooling operation.

Then, the concatenated features are passed into the following MST Blocks for deeper feature analysis and a parallelly organised average pooling. This layer downsamples the input signals and reduces the dimensionality. Moreover, implementing average pooling can also mitigate the problem of overfitting. Our proposed model employs average pooling since it considers every input value while generating output feature maps, illustrated in Fig 3. After the downsampling process, the downsampled features are passed to a 1 × 1 convolution to project information across channels and enable efficient low-dimensional embedding.

### 2.2. Incorporation of one-dimensional separable convolution

Most deep learning models in the HAR domain are dense and require heavy computations to achieve adequate performance. Besides requiring a longer time to converge, these models have a relatively slow inference time, which is unsuitable for real-world applications. Hence, our primary aim is to develop a lightweight HAR architecture that can perform efficient activity recognition. Instead of using the traditional one-dimensional (1D) convolutions, we adopt 1D separable convolutions. In 1D separable convolutions, the convolutional operations are split into two parts. Firstly, the input signals are fed into a depthwise separable convolution, where intermediate representations are generated by applying each convolutional kernel to every input channel. Then, the intermediate outputs are forwarded to the one-by-one pointwise convolution to integrate the outputs of depthwise separable convolutions.

The computational cost of a standard 1D convolution, $C_{cost}$, is as follows,


$$\begin{array}{c} C_{cost}=\;H_{k}\;.\;W_{k}\;.\;In\;.\;Out\;.\;H_{o}\;.\;W_{o}\; \end{array}$$ (1)

where $H_{k}$ and $W_{k}$ are the height and width of the convolutional kernels, In and Out are the numbers of input and output channels, and $H_{o}$ and $W_{o}$ are the height and width of the outputs. As for a 1D separable convolution, the cost of its computation for the same input and output size can be computed as below:


$$\begin{array}{c} {SC}_{cost}=\;H_{k}\;.\;W_{k}\;.\;In\;.\;H_{o}\;.\;W_{o}+In\;.\;Out\;.\;H_{o}\;.\;W_{o}\; \end{array}$$ (2)

With Equations (1) and (2), the reduction of the computation cost, ${Reduction}_{cost}$, using 1D separable convolution, can be calculated as follows:


$${Reduction}_{cost}=\;\frac{SC_{cost}}{C_{cost}}$$

$$\;=\;\frac{H_{k}\;.\;W_{k}\;.\;In\;.\;H_{o}\;.\;W_{o}+In\;.\;Out\;.\;H_{o}\;.\;W_{o}}{\;H_{k}\;.\;W_{k}\;.\;In\;.\;Out\;.\;H_{o}\;.\;W_{o}\;}\;$$

$$\begin{array}{c} \;=\frac{1}{Out}+\;\frac{1}{H_{k}\;.\;W_{k}}\; \end{array}$$ (3)

Let the input channel (i.e. In) is 9, the output channel (i.e. Out) is 32, the output size ${(H}_{o},\;W_{o})$ is (25,1), and kernel size ${(H}_{k}\;\times \;W_{k})$ is (5 × 1). The computational cost of a standard convolution would be 36000, whereas the computational cost of a separable convolution would be 8325. In summary, the computational cost of a separable convolutional operation is merely about one-fourth of a standard convolutional operation. This reduction rationalises using the 1D separable convolutions in the proposed Light-PTNet, as these layers are efficient in computation with fewer trainable parameters [29].

### 2.3. Modelling longer-term dependencies

Classifying human actions using smartphone signals is a time series classification problem. Moreover, human actions, especially dynamic activities (i.e., walking, climbing stairs, etc.) are quasiperiodic. So, temporal pattern analysis is vital in improving recognition accuracy. The input inertial signal varies according to the time for each activity. Therefore, the underlying discriminative pattern of the input signals should be retrieved by extracting these time-dependent features. In other words, temporal features provide rich information about motion signals. However, the main challenge is to model long-term dependencies in the features to preserve the activity-specific properties. The long-time series is assumed to contain long implicit patterns. Capturing long feature maps from the input series may provide abundant details about the inertial signals. Thus, retaining a long effective history can improve the classification accuracy, considering that the model can make a thorough decision with this rich information.

In order to model longer-term dependency, kernel elongation, dilation, and residual connection are always the common yet effective solutions [30]. Generally, the convolutional kernel sizes are increased to enlarge the receptive field of each kernel, which helps the model to extract longer patterns from inertial signals. The receptive field is the input series segment visible to a kernel at a time during the convolutional operation. Nevertheless, this technique has a major drawback. When the convolutional kernel sizes increase, the model parameters also increase exponentially. As a result, the classification model becomes more complex and prone to overfitting problems, especially if there is a small training set. Nevertheless, since our proposed model implements separable convolutions, integrating a larger convolutional kernel does not dramatically raise the overall parameters, leading to reasonable model complexity.

**Fig 4:**
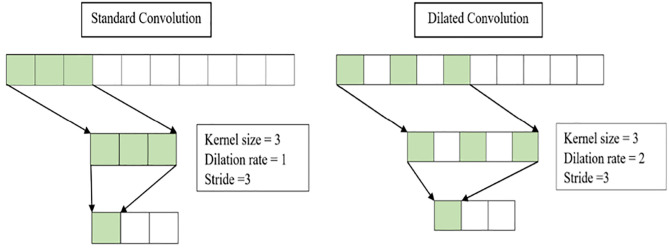
Standard convolution (left) and dilated convolution (right).

Besides kernel elongation, dilation is also an efficient solution to improve a convolutional kernel’s receptive field without elevating overall parameters. Dilations insert zeros between the kernel values, allowing each kernel to widen its field of view. Fig 4 shows the difference between a dilated and a standard convolution. The dilated convolution with a filter size of 3 has an identical visible region of the input space as the standard convolution with a filter size of 5. Thus, dilated convolutional kernels attain a larger field of view with fewer parameters. A convolutional kernel’s field of view can be manipulated by varying the kernel’s dilation rate, *d*. In the proposed model, dilations are applied at each layer within an MST Block, and the dilation rate is increased (e.g., $d=2^{0},\;2^{1},\ldots ,2^{L}$) at the subsequent layers. This hierarchical organisation of the dilated convolution enables the model to enlarge the receptive field further and gather richer and more crucial information about the input time series.

In order to retain a longer effective history of the input data, the proposed Light-PTNet incorporates a residual connection into each dilation level. The residual connection allows the inputs to skip the activation functions in each layer and pass them to the next layer. This property boosts the model training and prevents the model from the gradient vanishing and exploding problems, ensuring the model’s stability [12]. The residual connections in the proposed Light-PTNet consist of a one-by-one convolutional layer. Fig 2 illustrates the placement of the residual connection in the MST Blocks. Since the residual connection’s output will be concatenated with three LSTC Heads, one-by-one convolution will ensure that the residual connection’s output is the same size as each output of the LSTC Head.

### 2.4. Classification

**Fig 5:**
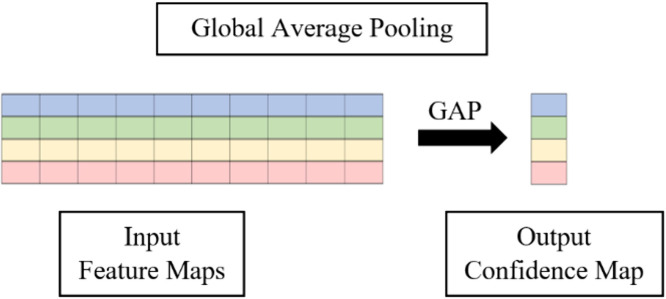
Global average pooling operation.

In traditional convolutional networks, the feature maps are processed further via fully connected dense layers. The feature maps are flattened to achieve the dimensions before passing them through the fully connected dense layers. However, this structure tends to negatively affect the overall model performance and lead to several challenges. For instance, the fully connected dense layers could substantially cause the model to have large parameters, increasing the complexity. This increased complexity, in turn, magnifies the likelihood of overfitting. Therefore, the Global Average Pooling (GAP) layer is employed in the proposed Light-PTNet instead of fully connected dense layers. Fig 5 illustrates a GAP operation. GAP operation allows the model to remove a large number of parameters from being trained. For instance, in the figure, the GAP layer takes in 4 channels of one-by-ten input and transforms them into the four channels of one-by-one output by averaging across the one-by-ten channel values of the input. This accelerates the overall training process. The parameter reduction makes the proposed model less susceptible to the overfitting problem. Unlike traditional fully connected dense layers, flattening is unnecessary for GAP as it produces the output in the desired shape. Moreover, GAP layers also do not require parameter optimisation.

The computed averaged feature maps, known as category confidence maps, are then forwarded to a softmax classifier to classify the input into respective activity classes. Firstly, the softmax classifier accepts the input sequence, x, and calculates a softmax score, $s_{k}(x)$, for each class, k, by computing the product of the transposed input, $x^{T}$ and each class’s dedicated parameter vector, $\theta^{\left(k\right)}$. The equation to calculate the softmax score, $s_{k}(x)$ is defined as follows:


$$\begin{array}{c} s_{k}(x)=x^{T}\theta^{\left(k\right)}\; \end{array}$$ (4)

The probability of an input ${\hat{P}}_{k}$, belongs to class k is estimated by applying the softmax function. The softmax function, σ() can be written as follows:


$$\begin{array}{c} {\hat{P}}_{k}=\;\sigma {\left(s_{k}(x)\right)}_{k}=\;\frac{e^{s_{k}(x)}}{\sum_{j=1}^{C}e^{s_{j}(x)}}\; \end{array}$$ (5)

where *C* is the number of classes. The estimated class probabilities are in the range between zero and one, and it is assumed that the target/predicted activity is the activity with the most significant estimated probability.

Besides the softmax function, the loss function is also crucial to any deep learning classifier, as this function helps optimise the model during training. Loss function measures how well predicted classes match the target classes. In Light-PTNet, categorical cross-entropy loss is implemented to calculate the error margin between the actual and the estimated value. The computed error is backpropagated throughout the model to improve future classification accuracy. The categorical cross-entropy function, CE is defined as:


$$CE\;=-\sum_{i}^{K}y_{k}^{\left(i\right)}\text{log}\left({{\hat{P}}_{k}}^{\left(i\right)}\right)$$ (6)

where $y_{k}^{\left(i\right)}$ is the ground truth of $i^{th}$ input belongs to class k and ${{\hat{P}}_{k}}^{\left(i\right)}$ is the predicted value of $i^{th}$ input belongs to class k.

## 3. Experimental setup and evaluation

### 3.1. Dataset description

Three publicly accessible smartphone-based HAR datasets, including UCI HAR, WISDM V1, and UniMiB SHAR are utilised for model validation. Anguita et al. collected smartphone signals from thirty volunteers aged 19–48 to build the UCI HAR dataset [16]. The volunteers wore smartphones (embedded with accelerometers and gyroscopes) around their waists while performing six activities: Walking, Walking Upstairs, Walking Downstairs, Standing, Sitting and Laying. The signal sampling rate is 50 ms^-2^. During pre-processing, noises and null values are removed from the input samples. Next, three groups of triaxial inertial signals, i.e., total acceleration, body acceleration and gyroscope, are vertically stacked to form nine channels. Then, the input signals are segmented into smaller sample windows with 128 data points using the sliding window technique.

WISDM V1 consists of triaxial data collected using smartphone accelerometers from thirty-six volunteers [31]. The smartphone is placed in the volunteer’s front pocket while performing the required six activities: Walking, Jogging, Upstairs, Downstairs, Sitting and Standing. The collected signal samples are pre-processed to remove null values. In WISDM VI, only one set of triaxial signals is collected from the accelerometer sensor: total acceleration. The sliding window technique splits the inertial signals into equally sized time windows with 200 data points.

UniMiB SHAR database is contributed by thirty volunteers aged 18–60. The volunteers perform seventeen activities, including activities of daily living (ADL) and falls, while having their smartphones in their front pants pockets. The dataset consists of triaxial signals from accelerometers. The authors group the samples into four sets according to the activity classes: (1) AF-17, (2) A-9, (3) AF-2 and (4) F-8 [32]. AF-17 group contains seventeen activity classes, including ADL and FALL. The A-9 group has only nine ADL classes. In the AF-2 group, all the ADL classes are considered as one class and all the FALL classes as another. Lastly, the F-8 group has only eight FALL activity classes. In this work, we merely use the AF-17 set to assess the proposed Light-PTNet’s performance. Similar to other database samples, the input data is cleaned before the segmentation. Next, the triaxial signals are stacked and segmented into sample windows, each with 151 data points. Table 1 summarises the evaluation datasets utilised in this research study.

**Table 1:** Description of UCI HAR, WISDM V1 and UniMiB SHAR.

Dataset	UCI HAR	WISDM V1	UniMiB SHAR
Sensor	Accelerometer and Gyroscope	Accelerometer	Accelerometer
Subjects	30	36	30
Segment Size	128	200	151
Sampling Rate	50 ms-2	20 ms-2	50 ms-2
Channel Size	9	3	3
Activities (Class Label)	Walking, Upstairs, Downstairs, Sitting, Standing and Laying	Jogging, Walking, Upstairs, Downstairs, Sitting and Standing	Only the AF-17 set is used with 17 classes: StandingUpFS, StandingUpFL, Walking, Running, GoingUpS, Jumping, GoingDownS, LyingDownFS, SittingDown, FallingForw, FallingRight, FallingBack, HittingObstacle, FallingwithPS, FallingBackSC, Syncope and FallingLeft
Training Testing Split	21 training users: 9 testing users	26 training users: 10 test users	21 training users: 9 testing users
Validation Split	20% of the training set	20% of the training set	20% of the training set
Validation protocol	Subject Independent Protocol	Subject Independent Protocol	Subject Independent Protocol

### 3.2. Model configuration

The proposed Light-PTNet is developed using Tensorflow and Keras on a desktop with Intel® Core™ i9-12900K CPU with 2.20 GHz, 32GB RAM, NVIDIA GeForce RTX 3080 Ti and 12GB memory. The training epochs for the proposed Light-PTNet are 100 for all three datasets. Moreover, the Reduce Learning Rate on Plateau function is implemented to enable dynamic learning rates during training. This function reduces the learning rate when the proposed model’s validation loss does not decrease, leading to faster model convergence. The hyperparameter configuration of the proposed Light-PTNet is recorded in Table 2. The hyperparameter values were determined through a systematic tuning process during the architecture analysis phase. A range of values was chosen for each hyperparameter based on previous literature and domain knowledge. The optimal values identified from this process were then used in subsequent experiments. The experiments conducted to support these configuration settings are detailed in Section 4.1.

**Table 2:** The hyperparameter settings of the proposed Light-PTNet.

Hyperparameters	UCI HAR	WISDM V1	UniMiB SHAR
Input Dimension	(128,9)	(200,3)	(151,3)
Batch Size	32	64	32
Number of Blocks	2	2	3
Number of Filters	32	40	32
Filter Size	4, 8 and 12	4, 8 and 12	4, 8 and 16
Dilation Rate	1, 2, and 4	1, 2, and 4	1, 2, and 4
Stride	1	1	1
Dropout Rate	0.05	0.05	0.10
Number of Epoch	100	100	100
Initial Learning Rate	0.001	0.001	0.001
Reduce Learning on Plateau Function.	Minimum learning rate = 0.0001 Mode = validation loss Patience = 3	Minimum learning rate = 0.0001 Mode = validation loss Patience = 3	Minimum learning rate = 0.0001 Mode = validation loss Patience = 3
Optimiser	Adam	Adam	Adam
Loss function	Categorical cross-entropy	Categorical cross-entropy	Categorical cross-entropy

## 4. Result and discussion

### 4.1. Network architectural analysis

Architectural analysis is performed on certain proposed model components to study their importance in classification performance. UCI HAR dataset is used to determine the optimal structure of Light-PTNet. This analysis examines the effects of convolution types, the causality of the convolutions, the effects of dilations, and the significance of GAP and average poolings in the proposed model.

Firstly, two types of convolutions (i.e., standard 1D convolution and 1D separable convolution) are examined. Generally, a model with a large number of learnable parameters would extract more significant features from the input data, achieving higher classification accuracy. However, having gigantic model parameters will increase model complexity and slow down the model convergence during training. Furthermore, this condition may make the model more susceptible to overfitting problems. As shown in Table 3, we notice that the proposed model using 1D separable convolutions possesses only 0.052 million (M) learnable parameters yet achieves a high accuracy of 98.03%. Conversely, the model using standard 1D convolutions has approximately five fold the number of parameters but attains a lower accuracy by ~2%.

**Table 3:** Performance of Light-PTNet with different types of convolutions.

Convolution Type	Number of Parameters (*Million, M*)	Precision	Recall	F1 Score	Test Accuracy (*%*)
1D separable convolution	0.052	0.9819	0.9804	0.9808	98.03
Standard 1D convolution	0.286	0.9690	0.9683	0.9680	96.84

The second experiment is designed to observe the causality’s effect on the proposed Light PTNet’s performance since our model is inspired by the TCN architecture, which utilises causal padding. Table 4 presents the proposed Light-PTNet’s performance. The model with causal convolution, unfortunately, performs poorer than the one without. As a result, the model’s test accuracy improves by ~3% when the aspect of causality is removed.

**Table 4:** Performance of Light-PTNet with different causality settings.

Causality	Precision	Recall	F1 Score	Test Accuracy (*%*)
Causal Convolution	0.9618	0.9582	0.9585	95.79
Without Causal Convolution	0.9819	0.9804	0.9808	98.03

The third experiment is performed by manipulating the dilation rates of the 1D separable convolutional layer in LSTC Heads. This experiment aims to understand the impact of increasing dilation rates on the proposed Light-PTNet’s performance. Table 5 records the model performance with different dilation settings. The results show that the total learnable parameters increase with higher dilation rates. The classification performances of the proposed model improved by approximately 4% for the first three dilation settings, indicating that the convolutional kernels can capture long underlying temporal patterns from the input signals. However, the proposed Light-PTNet’s performance degrades when the dilation rate is set to 1, 2, 4 and 8. This deterioration may be due to the overexpansion of the convolutional kernel, causing the model to lose its ability to capture fine-grained temporal patterns from inertial signals.

**Table 5:** Performance of Light-PTNet with different dilation settings.

Dilation Rate	Number of Parameters (*Million, M*)	Precision	Recall	F1 Score	Test Accuracy (*%*)
None	0.017	0.9423	0.9442	0.9431	94.30
1 and 2	0.036	0.9627	0.9616	0.9612	96.17
1, 2 and 4	0.052	0.9819	0.9804	0.9808	98.03
1, 2, 4 and 8	0.074	0.9776	0.9757	0.9762	97.56

Next, the significance of the GAP layer in the proposed Light-PTNet is studied. Two variations of the proposed model are designed: one with a GAP layer and one with a fully connected dense layer. Table 6 presents the experimental results. The model with a fully connected dense layer is more complex and denser than the model with a GAP layer with 26-fold trainable parameters. The fully connected dense layer drastically raises the trainable parameters, causing higher model complexity and computational costs. The increased complexity makes the model more vulnerable to overfitting, leading to performance degradation. Unlike fully connected dense layers, the model with a GAP layer achieves a higher classification accuracy of 98.03% with only 0.052 million parameters. Furthermore, the GAP layer does not require additional parameter optimisation.

**Table 6:** Performance of Light-PTNet with/without GAP.

Implementation of GAP	Number of Parameters (*Million, M*)	Precision	Recall	F1 Score	Test Accuracy (*%*)
No (Fully Connected Dense Layer)	1.336	0.9592	0.9571	0.9574	95.69
Yes	0.052	0.9819	0.9804	0.9808	98.03

Table 7 records the proposed Light PTNet’s performance with different pooling settings: (1) without pooling, (2) with average pooling in each MST Block and (3) with average pooling in each MST Block except the first block. As expected, the model without average pooling performs the worst. On the other hand, incorporating average pooling in each MST Block enhances the overall system accuracy by ~2% while reducing the generated feature representations’ dimensionality. The performance of Light-PTNet further increased by ~1% when the average pooling layer was removed from the first MST Block.

**Table 7:** Performance of Light-PTNet with different pooling settings.

Pooling	Precision	Recall	F1 Score	Test Accuracy (*%*)
None	0.9603	0.9586	0.9582	95.86
Average Pooling in each block	0.9755	0.9738	0.9741	97.39
Average pooling in each block except the first block	0.9819	0.9804	0.9808	98.03

### 4.2. Performance evaluation and comparison

The proposed Light-PTNet’s performance is evaluated and compared against other HAR methods across three databases. Furthermore, model complexity comparisons between the proposed Light PTNet and other lightweight deep learning architectures are also presented in the following subsection. All the existing methods selected for performance comparison also implement a subject-independent validation procedure where the test set contains samples from different subjects, similar to our proposed Light-PTNet, to ensure fair comparison.

#### 4.2.1. UCI HAR.

**Fig 6:**
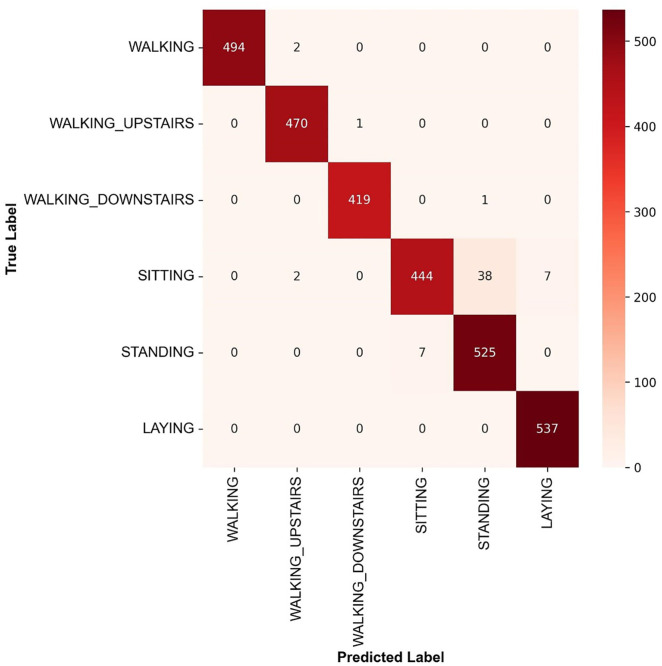
Confusion matrix of Light-PTNet in UCI HAR.

This work observes a subject-independent testing procedure to validate Light-PTNet’s performance. This procedure prevents the training and testing sets from having samples from the user. UCI HAR is segregated into three subsets, where the training set contains samples from the first 21 users, 20% of which are used for validation, and the samples from the remaining nine users are placed in the test set. Signal pre-processing is crucial in pattern recognition. Table 8 records the proposed Light-PTNet’s classification performance with and without data pre-processing. In this work, we normalise the captured inertial signal by transforming the signal data to the range of [0 1]. The proposed Light-PTNet with normalised inputs attained higher accuracy, improving ~1% over that without pre-processing. Fig 6 illustrates the confusion matrix of Light-PTNet. Generally, the proposed model obtains a very low misclassification rate in every activity class except for the *sitting* class. Some of the sitting class samples are misclassified as the *standing* class, and some as the *laying* class. The reason behind these misclassifications is the low interclass variance among these three activity classes due to their static pattern nature. The training accuracy of the proposed Light-PTNet is 100% and the test accuracy slightly drops due to the test set containing new unseen samples from different subjects.

**Table 8:** Performance of Light-PTNet with different pre-processing settings in UCI HAR.

Signal pre-processing	Test Accuracy (%)
Without data normalisation	97.01
Data normalisation	98.03

The recognition accuracy performances of the proposed Light-PTNet and the other HAR architectures are recorded in Table 9. The proposed Light-PTNet dominated the existing HAR solutions by achieving an accuracy of 98.03%. We notice that the proposed Light-PTNet surpasses most of the handcrafted feature-based methods by a large margin. This again validates its ability to generalise to unseen data. Furthermore, the proposed Light-PTNet’s performance exceeded existing deep learning architectures, including CNN, recurrent, and TCN models. The superior performance could be due to the multiscale feature extraction capability of the Light-PTNet, which facilitates the extraction of richer information about the motion signals. Several CNN models, such as CNN with Statistical features [10], Selective Kernel CNN [33], HS-ResNet [34] and ResNet with SelectConv [21], show promising recognition performances. However, these models require either manual feature engineering or complex and deep analysis with gigantic parameters in order to accomplish such classification performances.

**Table 9:** Accuracy comparison for subject-independent UCI HAR.

Methods	Test Accuracy (%)
Statistical features + Continuous HMM [18]	91.76*
Statistical features + HMM Ensemble [35]	83.51*
Statistical features + RF [17]	78.00*
WSTM [36]	90.33*
Evidential Reasoning with Hesitant Fuzzy Belief Structures [37]	95.41*
Predsim [38]	96.98*
Attention-induced multi-head CNN [39]	95.37*
CNN [40]	93.21*
CNN + C3 [20]	96.96*
Selective Kernel CNN [33]	97.21*
CNN with smaller filters [11]	96.27*
Baseline+SelectConv [21]	96.77*
ResNet+SelectConv [21]	97.28*
CNN+Statistical features [10]	97.63*
HS-CNN [34]	96.77*
HS-ResNet [34]	97.38*
Deep Residual Bidirectional LSTM [12]	93.60*
Bidirectional LSTM [23]	93.79*
CNN LSTM [13]	92.13*
Dilated TCN [14]	93.80*
Encoder-Decoder TCN [14]	94.60*
Proposed Light-PTNet	98.03

* Results extracted from the respective articles

#### 4.2.2. WISDM VI.

**Fig 7:**
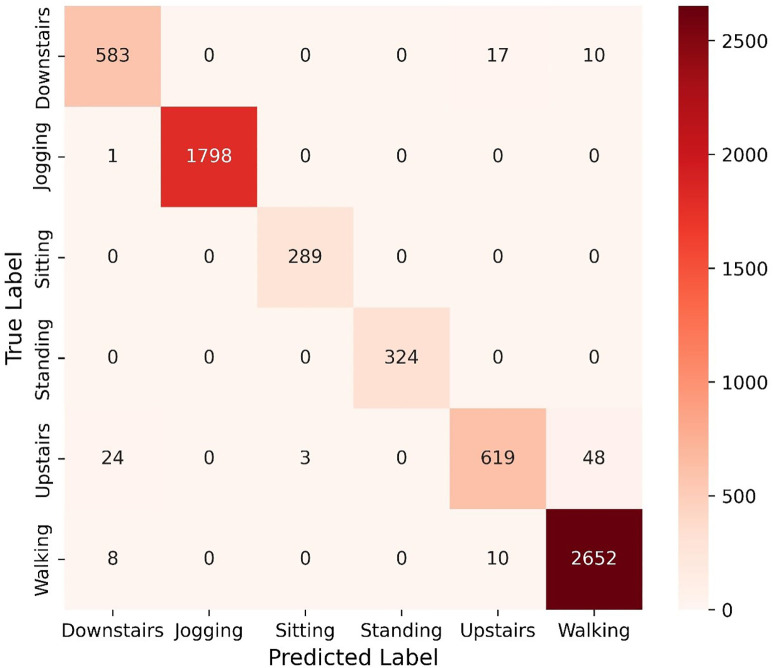
Confusion matrix of Light-PTNet in WISDM V1.

In this work, WISDM V1 is evaluated using a subject-independent protocol. Data samples from randomly selected twenty-six volunteers are utilised as training samples, 20% of which are selected as validation samples, and the samples from the remaining ten volunteers are utilised for testing. The proposed Light-PTNet is trained with normalised and unnormalised input data separately to understand whether the data normalisation process improves model accuracy in the WISDM V1 dataset. Table 10 shows that implementing data normalisation improved the proposed Light-PTNet’s performance by approximately 2%. This again signifies the significance of data normalisation in pattern recognition. Fig 7 illustrates the confusion matrix of the proposed model. From the confusion matrix, it is observed that the *upstairs* class had the most misclassifications. The proposed model misclassified 24 samples of the *upstairs* class as the *downstairs* class and another 48 samples of the *upstairs* class as the *walking* class. Light-PTNet attained an accuracy of 96.79% during the training process and achieved a slightly higher accuracy of 97.02% during testing.

**Table 10:** Performance of Light-PTNet with different pre-processing settings in WISDM V1.

Signal pre-processing	Test Accuracy (%)
Without data normalisation	95.46
Data normalisation	97.02

Table 11 records the classification accuracy of Light-PTNet and several state-of-the-art HAR algorithms. Our proposed model acquired 97.02% accuracy and outperformed existing methods, including handcrafted feature-based and deep learning-based techniques, by a large margin. The proposed Light-PTNet outperforms the handcrafted feature-based methods by approximately 14% due to its competence in capturing underlying spatiotemporal features. Since the proposed model does not rely on prior knowledge, it is less susceptible to biases, thereby improving its generalisation ability. Additionally, Light-PTNet operates automatically, obviating the necessary manual feature engineering as required by handcrafted methods. The statistical features + CNN model [8] and Deep Convolutional Neural Network (DCNN)+Statistical features model [19] attain the second-highest classification accuracy among deep learning models after our proposed architecture. Nonetheless, the proposed Light-PTNet’s performance is still superior to those models as it achieves 97.02% with an extremely low number of trainable parameters compared to the DCNN model (will be discussed in the Model Computational Complexity section).

**Table 11:** Accuracy comparison for subject-independent WISDM V1.

Methods	Test Accuracy (%)
Statistical features + RF [7]	83.46*
Statistical features + RF [41]	83.35*
Statistical features + Dropout Classifiers [7]	85.36*
Statistical features + CNN [10]	93.32*
Dilated and Strided CNN [42]	88.27*
Data Augmentation + Two Stage End-to-End CNN [43]	84.60*
Deep Convolutional Neural Network (DCNN)+Statistical features [19]	94.18*
CNN [44]	84.00*
Compatibility-based classifier personalisation [45]	80.60*
Proposed Light-PTNet	97.02

* Results extracted from the respective articles

#### 4.2.3. UniMiB SHAR.

**Fig 8:**
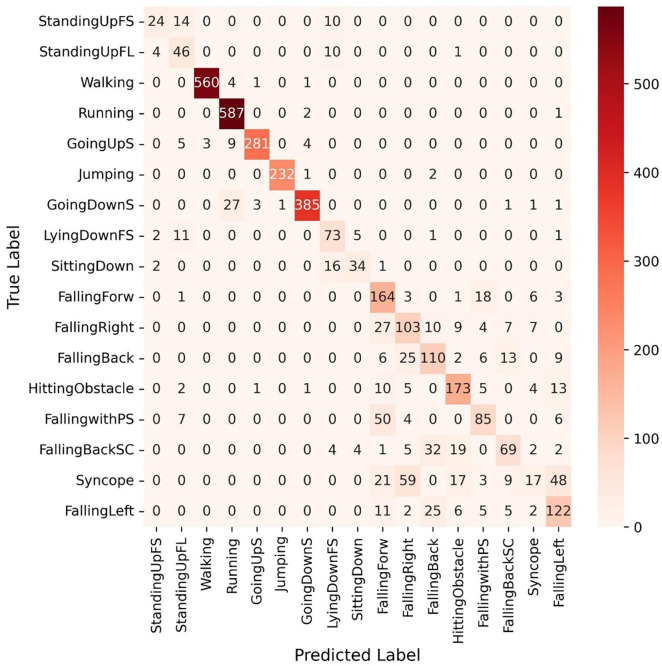
Confusion matrix of Light-PTNet in UniMiB SHAR.

The efficiency of the proposed Light-PTNet on UniMiB SHAR is assessed using a subject-independent procedure. All the samples from 21 users are placed in the training set, 20% of the training samples in the validation set, and the samples from the remaining users in the test set. Similar to the previous datasets, the proposed Light-PTNet is tested with and without data normalisation. Table 12 shows the proposed Light-PTNet’s performance under different pre-processing settings. As observed in the previous finding, applying data normalisation improves the proposed model’s performance by approximately 3%. The confusion matrix of Light-PTNet is illustrated in Fig 8. From the confusion matrix, we can notice that the FALL-related activity classes were more frequently misclassified compared to the ADL activity classes. This suggests that the proposed model is suboptimal for distinguishing between the FALL activities, which may be due to the low interclass variance. The training accuracy of the proposed Light-PTNet is 100%. However, the test accuracy drops as the model was not able to accurately predict the FALL classes.

**Table 12:** Performance of Light-PTNet with different pre-processing settings in UniMiB SHAR.

Signal pre-processing	Test Accuracy (%)
Without data normalisation	77.62
Data normalisation	81.58

Table 13 records the accuracy performance of the proposed Light-PTNet and the other popular HAR models. The proposed model attained 81.58% accuracy on the test set and outperformed the existing models by obtaining ~1.5% to ~7% higher accuracy. This indicates the efficiency of the proposed model in extracting rich information, such as temporal features, from the input sequences for classification. Moreover, enlarging the convolutional kernel’s receptive field via the dilations and residual connections ensures that longer-term dependency is preserved throughout the proposed network. These structures allow extensive time-dependent information extraction of the motion signals. Hence, the proposed network generalises well and makes better predictions on the test set. Predsim ResNet [46] and ResNet-DFC [47] models attain the second-best classification performance on this dataset with only a minor difference of ~1.5%. However, the proposed Light-PTNet excels over these models in terms of efficiency. Our proposed architecture achieves this high performance with minimal learnable parameters (i.e., 0.08 million parameters). This is considerably fewer parameters compared to Predsim ResNet [46] and ResNet-DFC [47] models (will be discussed in the section Model Computational Complexity).

**Table 13:** Accuracy comparison for subject-independent UniMiB SHAR.

Methods	Test Accuracy (%)
HS-CNN [34]	77.26*
HS-ResNet [34]	79.19*
Asymmetric Residual Neural Network [48]	76.39*
Predsim [38]	78.07*
Predsim CNN [46]	78.11*
Predsim ResNet [46]	80.33*
CNN-DFC [47]	79.11*
ResNet-DFC [47]	80.02*
CNN with smaller filters [11]	74.46*
Baseline+SelectConv [21]	77.26*
ResNet+SelectConv [21]	78.25*
CNN + C3 [20]	75.16*
CNN-TAMA [49]	77.01*
DanHAR [50]	79.03*
Selective Kernel CNN [33]	76.84*
Proposed Light-PTNet	81.58

* Results extracted from the respective articles

### 4.3. Model computational complexity

For real-world applications, the computational complexity of a classification model is essential. For a system model intended for real-world application, computational complexity ought to be considered acutely due to the time constraint for model building and prediction, available resource limitations, etc. In this work, three evaluation criteria are chosen to measure the computational complexity of the proposed model. The evaluation criteria are as follows:

i *Number of model parameters*: total trainable parameters available in the model.
ii *Model size*: the size of the fully trained model.
iii *Training time*: total number of epochs required for model convergence during the training phase.

The computational complexity of Light-PTNet across three databases (i.e., UCI HAR, WISDM VI and UniMiB SHAR) is recorded in Table 14. The table shows that the proposed model is computationally lightweight, with less than 0.1 million parameters across all three databases. Additionally, the proposed model is compact and only needs 100 *epochs* to converge while attaining an encouraging recognition performance. Conversely, the traditional CNN model with smaller kernels contains over 1 million model parameters and requires 500 epochs to converge [11].

**Table 14:** Computational complexity of Light-PTNet.

Dataset	Number of Model Parameters (*Million, M*)	Model Size (*Mb*)	Training Time (*epochs*)
UCI HAR	0.052	1.4	100
WISDM V1	0.077	1.7	100
UniMiB SHAR	0.082	2.1	100

Table 15 shows the computational complexity, in terms of the number of model parameters and training time, of the other HAR deep learning models, including lightweight models. The proposed Light-PTNet demonstrates a remarkable performance with the lowest trainable parameters and shortest training time compared to the other light models across all three databases. Among the models, Resnet-DFC [47], CNN with smaller filters [11], ResNet with SelectConv [21], and DanHAR [50] possess the largest number of model parameters and the longest training time for model convergence. On the other hand, Predsim Resnet [46] has the lowest model parameters among the other lightweight models.

**Table 15:** Computational complexity comparison between the proposed Light-PTNet and existing deep learning models across three databases.

Deep Learning Models	Model Parameter (Million)	Training Time (epochs)
**UCI HAR Database**		
HS-CNN [34]	0.460*	200*
HS-ResNet [34]	0.670*	200*
Attention-induced multi-head CNN [39]	1.511*	240*
CNN with smaller kernels [11]	1.300*	500*
Baseline+SelectConv [21]	0.330*	200*
ResNet+SelectConv [21]	0.840*	200*
CNN + C3 [20]	0.342*	200*
Selective Kernel CNN [33]	0.450*	500*
Predism [38]	0.350*	500*
The proposed Light-PTNet	0.052	100
**WISDM V1 Database**		
DCNN+Statistical features [19]	1.383*	100*
The proposed Light-PTNet	0.077	100
**UniMiB SHAR Database**		
HS-CNN [34]	0.510*	200*
HS-ResNet [34]	0.720*	200*
Predsim [38]	0.550*	500*
Predsim Resnet [46]	0.110*	–
CNN-DFC [47]	1.670*	150*
Resnet-DFC [47]	6.670*	150*
CNN with smaller kernels [11]	5.800*	500*
Baseline+SelectConv [21]	1.550*	200*
ResNet+SelectConv [21]	3.620*	200*
CNN + C3 [20]	1.524*	200*
CNN-TAMA [49]	0.350*	200*
DanHAR [50]	2.400*	500*
Selective Kernel CNN [33]	0.540*	500*
The proposed Light-PTNet	0.082	100

* Results extracted from the respective articles

Besides, several deep learning models such as HS-CNN [34], Baseline with SelectConv [21], CNN with C3 module [20], Selective Kernel CNN [33], Predism [38], and CNN-TAMA [49] contain a moderate amount of learnable parameters. However, these models show poorer recognition performances (refer to Tables 9 and 13). As aforementioned, DCNN model [19] on WISDM V1 and Predsim ResNet [46] and ResNet-DFC [47] models on UniMiB SHAR achieve the second-highest classification accuracies on the respective datasets (see Tables 11 and 13). In WISDM V1, our proposed Light-PTNet is more efficient than DCNN model, attaining a higher classification accuracy with merely 0.077 million parameters, approximately 17 times fewer parameters than DCNN model. Additionally, Light-PTNet model only requires 100 *epochs* to converge. Similarly, Resnet-DFC [47] model contains 6.670 million parameters, 81 times more than the proposed Light-PTNet, in order to achieve the accuracy of 80.02% on UniMiB SHAR. Furthermore, it is worth noting that our proposed model is still considered superior to Predsim ResNet [46] model on UniMiB SHAR, as our proposed model achieves slightly higher accuracy (~1.25%) with relatively low parameters (0.082 million) compared to Predsim ResNet [46] model (0.110 million parameters).

## 5. Conclusion

A lightweight TCN variant, coined as Light-PTNet, is proposed in this work for human activity recognition. Like other deep learning models, the proposed model requires no manual feature engineering and complex pre-processing. The key advantage of our model resides in its lightweight computation, incorporated with multiscale feature extractions that contribute to promising recognition performance. The parallel organisation of LSTC Heads within the MST Block at each dilation level captures temporal features at different scales, facilitating multiscale feature extraction. The concatenation of the generated feature maps provides richer information, and this helps the proposed model to generalise better. Another characteristic of the proposed model is its capability to retain a longer-term dependency. Longer convolutional kernels are used in the model to extract longer temporal features. Dilations and residual connections are implemented to preserve a longer effective history of the input signals. Besides preserving information, the residual connection can prevent the vanishing and exploding gradient problem. The feasibility of Light-PTNet is validated on three publicly available datasets (i.e., UCI HAR, WISDM VI and UniMiB SHAR). Empirical results affirm the proposed model’s efficiency in human activity recognition, exhibiting promising recognition performance (i.e., 98.03% accuracy on UCI HAR, 81.58% on UniMiB SHAR and 97.02% on WISDM V1) with fewer learnable parameters. Besides that, Light-PTNet also demonstrates a relatively short training time.
